# Levels of Physical Activity in Spanish Asthmatics: A Cross-Sectional Study

**DOI:** 10.3390/medicina56120643

**Published:** 2020-11-25

**Authors:** Sheila Sánchez Castillo, Lee Smith, Arturo Díaz Suárez, Guillermo Felipe López Sánchez

**Affiliations:** 1 Faculty of Sports Sciences, University of Murcia, 30720 Murcia, Spain; ardiaz@um.es; 2 Cambridge Centre for Sport and Exercise Science, Anglia Ruskin University, Cambridge CB5 8DZ, UK; Lee.Smith@aru.ac.uk

**Keywords:** respiratory disease, physical exercise, epidemiology, tobacco, body mass index, alcohol

## Abstract

*Background and objectives:* 339 million people in the world suffer from asthma. Regular physical activity (PA) could help in its control. Therefore, the aim of this research was to determine the level of PA in Spanish people with asthma considering variation by, age, sex, education, marital status, living together, smoking habits, alcohol intake and body mass index (BMI). *Materials and Methods:* 1014 Spanish people from 15 to 69 years were included in the study. Data of the Spanish Health Survey (year 2017) were analysed. PA levels were measure with the international physical activity questionnaire short version (IPAQ-SF). PA was categorized as low, moderate and high, and analyzed by sample characteristics. Mann-Whitney U test, Kruskall Wallis H and crosstabs were used to calculate statistical significance (*p* < 0.05). *Results:* On average, Spanish asthmatics engaged in a weekly volume of 2228.9 metabolic equivalent of task (MET)·min/week. Males revealed significantly higher PA than females (2516.8 vs. 2019.5 MET·min/week; *p* = 0.005), younger participants (<30 years) compared to people aged 30–60 years and older than 60 years (2699.0; 2243.2; 1619.3 MET·min/week; *p* < 0.001) and those with tertiary level of education than those without secondary (2368.3 vs. 2168.3 MET·min/week; *p* = 0.001). Level of PA was lower in those married (*p* = 0.001) and/or living together (*p* = 0.010). Alcohol consumers showed a higher level than the participants who did not drink (2378.3 vs. 1907.9 MET·min/week; *p* = 0.001), but no significant differences were found within current, past and never smokers (*p* = 0.890). Obese asthmatics engaged in less PA than their normal weight and overweight peers (*p* < 0.001). Overall, moderate level was significantly the most frequent (47.7%), but 31.6% showed a low level. *Conclusions:* Three out of ten Spanish people with asthma do not achieve PA recommendations, so PA programs should be executed to make people aware of its benefits in asthma control, focusing on those groups with lower PA levels.

## 1. Introduction

Asthma is a common and important chronic disease that involves people of all ages [1]. Globally, 339 million people suffer from asthma [2]. In adults, the overall prevalence diagnosed is estimated to be 4.3% [3]. In Spain, an epidemiological study of chronic obstructive pulmonary disease (IBERPOC Project), found a 4.9% prevalence of asthma in Spanish adults aged from 40 to 69 years [4], but according to data of the Spanish Health Survey (year 2017), the prevalence of asthma in Spanish people aged 15 to 69 years was found to be slightly higher, at around 6% [5].

Asthma is a chronic condition that appears with chronic airway inflammation, whose main symptoms are wheeze, chest tightness, shortness of breath, variable expiratory airflow limitation and cough [1]. All these aspects, together with the fear of having exercise-induced bronchoconstriction (EIB), could have a negative effect on levels of physical activity (PA) in people with asthma [6]. Thus, some studies have determined that asthmatics frequently engage in a lower amount of PA than people without asthma [6,7]. Nevertheless, constant practice of PA helps to prevent several chronic diseases [8]. In asthmatics, regular PA aids in the control of asthma [9,10], which consequently reduces the risk of asthma crisis [1]. Different investigations suggest that a usual amount of PA reduces the symptoms of asthma [11,12]: airway responsiveness [13], EIB (demonstrated in children) [10] and the risk of asthma exacerbations [13]. Moreover, it has also been demonstrated that PA improves exercise capacity [14,15] and quality of life [10,13] in people suffering from asthma. Regarding lung function, there is no agreement about the benefits of PA. Carson et al. found that PA had no significant impact on lung function [14], but Eichenberger et al. found improvements in Forced Expiratory Volume in the first second (FEV_1_) in asthmatics who engaged in exercise training [15].

It is important to underline that a higher number of urgent primary care consultations because of asthmatic exacerbations are related to a higher economic burden [16]. An important part of the cost of asthma is associated to urgent admissions, hospitalization and mortality [17]. Thus, uncontrolled asthma has significantly high costs [18], which could be reduced by improving disease control. In a recent systematic review, the literature analysed suggested that more moderate-vigorous intensity PA was associated with a better control of asthma [4]. Moreover, another systematic review has proposed that people involved in more PA may have less risk of developing asthma [13], which will contribute to reducing health costs.

Nevertheless, the vast majority of studies on PA and asthma have been carried out in populations of children [19,20]. In adults, there is lack of knowledge about levels of PA in asthmatic populations. Some studies have investigated PA in adults with asthma in relation to BMI [21,22] and age [23]. Another investigation found that men, younger and normal weight asthmatics did more PA and had better quality of life than women, older people and overweight or obese adults with asthma [24]. To the authors’ knowledge, there is no literature regarding PA levels in asthmatics according to marital status, cohabiting, education level, smoking habits and alcohol consumption, which have all been shown to be potential correlates of PA in the general adult population [25,26].

It is hypothesized that Spanish people with asthma will participate in less PA than their peers without asthma. It is also hypothesized that PA will be lower in women, in older adults, in people with lower education, in tobacco and alcohol consumers and in obese people.

Hence, the aim of the present investigation was to evaluate the PA levels among Spanish people with asthma and to study possible variations by sex, age, education, marital status, cohabiting, tobacco habits, alcohol intake and BMI.

## 2. Materials and Methods

### 2.1. Study Design

The design of this research was cross-sectional, and it is presented according to the Strengthening the Reporting of Observational studies in Epidemiology (STROBE) checklist (https://www.strobe-statement.org) [27].

### 2.2. Setting

We used data from the last Spanish Health Survey (year 2017). All methods used in this survey have been previously made public [28]. In short, this survey was carried out between October 2016 and October 2017 throughout the Spanish territory. Data was collected by using stratified sampling in three stages. Firstly, census sections were taken into consideration. Then family dwellings were selected by systematic sampling, which allowed self-weighting samples in each stratum. Finally, an adult (aged 15 or over) from each dwelling was chosen to complete the Adult Questionnaire by using the random Kish method. Data were collected by the computer-assisted personal interviewing (CAPI) method, administrated in the participant’s homes. The present research was developed following the Declaration of Helsinki of the World Medical Association. No ethical approval was required because the data used are anonymous and public [28]. Nevertheless, the Ethical Research Committee of the University of Murcia (Spain) approved these secondary analyses (ethical code: 2403/2019; approval date: 30/04/2019).

### 2.3. Participants

The sample was composed of 1014 Spanish people with asthma (587 women). Inclusion criteria were: (1) positive response to the question: “Have you ever been diagnosed with asthma?” and (2) being 15 to 69 years old. Participants of at least 70 years were not included in this study, because they had not answered the questions of the International Physical Activity Questionnaire (IPAQ) short form. This questionnaire was firstly purposed and tested for monitoring PA in adults (15 to 69 years), so until further advancement and testing is carried out in younger and older age groups, its use is not recommendable for those age groups [29].

### 2.4. Variables

The survey included sociodemographic questions (age, sex, last education level completed, marital status, smoking habits and alcohol consumption), physical characteristics (height and weight) and quantity of PA. These sociodemographic variables were chosen considering data available in the survey and previously identified correlates of PA in the general adult population [25,26].

#### 2.4.1. Physical Activity

The instrument used to determine participants’ PA level was the IPAQ short form. Total weekly amount of PA was calculated according to the method for computation of metabolic equivalent of task (MET)·minutes/week, previously established in the IPAQ instructions for analysis and data processing of the questionnaire [29]. Then, PA level was classified as: low (<600 MET·min/week), moderate (≥600 MET·min/week) and high (≥3000 MET·min/week), following the same guidelines. This questionnaire has acceptable validity (ρ = 0.30, 95% CI: 0.23–0.36) and reliability (Spearman’s ρ = 0.81, 95% CI: 0.79–0.82) [30]. It has also been validated in Spain (r = 0.277; *p* < 0.05; 75% of specificity and sensibility; k = 0.33).

#### 2.4.2. Sociodemographic Variables

Age was divided into three groups: less than 30 years, from 30 to 60 years, and 60 or more years, according to accepted international classifications of adults and older adults [31,32]. Education was grouped based on the highest level completed following the Spanish Classification of Education Levels as Level A (≤first period secondary), level B (second period secondary and post-secondary (not tertiary), and Level C (tertiary). Marital status was categorized as married and not married (single/widow/divorced/separated). Cohabiting was treated as a dichotomous variable: yes, if they are living together as a couple, or no. Alcohol intake was also categorized as yes/no, considering no consumption if they had never drunk alcohol or if they had never drunk alcohol in the last 12 months. Smoking habits were divided into three groups: those who had never smoked, those who had smoked before but did not smoke currently, and those who smoked currently [33]. In accordance to the methodology determined by the Spanish Health Survey [28], BMI was classified as follows: underweight (<18.5 kg/m^2^), normal weight (18.5–24.9 kg/m^2^), overweight (25–30 kg/m^2^) and obesity (>30 kg/m^2^).

### 2.5. Data Analysis

Sample characteristics were described by frequency and percentage. To check the normality of the data, chi-squared tests were used for qualitative variables and Kolomogorov-Smirnov for quantitative variables. All the studied variables had a nonparametric distribution, except marital status. Descriptive statistics were also used to describe PA level (MET·min/week) of subjects by gender, age, education, marital status, tobacco consumption, alcohol intake and BMI. Statistical significance was tested with the Mann-Whitney U test (gender, education, marital status, alcohol intake) and the Kruskal-Wallis H test for polytomous variables (age, tobacco, BMI) with Bonferroni correction for pairwise comparisons.

To determine significant differences in PA classification between the groups analysed, crosstabs with chi-squared and adjusted residual values were used. In those variables in which chi-squared tests had significant results, the *p*-value of each box was calculated based on the adjusted residual value in order to identify between which groups were the differences significant. Moreover, Pearson Correlation was employed to analyse the association between PA and age. The risk for low PA levels (outcome) was calculated by odds ratio with a confidence interval of 95% (OR; 95%CI); the multivariate regression model was adjusted for age, BMI, sex, education, cohabiting, marital status, tobacco and alcohol consumption. The effect size was calculated using eta squared for the Mann-Whitney U test, epsilon squared for the Krukal Wallis H test and Cramer’s V for the chi squared test. Eta squared was calculated by using the following formula: ɳ2 = $Z^{2}/\left(n-1\right)$, epsilon squared was calculated as $\frac{H}{\left(n^{2}-1\right)/\left(n+1\right)}$ and Cramer’s V by using this formula: V = $\sqrt{\chi^{2}/\left(n\times \min\left(k-1,c-1\right)\right)}$ [34]. Effect size of eta squared was classified as small (0.01), medium (0.06) and large (0.14); effect size of epsilon squared was classified as negligible (0.00–0.01), weak (0.01–0.04), moderate (0.04–0.16), relatively strong (0.16–0.36), strong (0.36–0.64) and very strong (0.64–1.00); effect size of Cramer’s V was classified as small (0.1), medium (0.3) and large (0.5).

Statistical analyses were performed using the Statistical Package for Social Sciences version 23 (SPSS, International Business Machines Corporation, Armonk, NY, USA). Statistical significance was set at *p* < 0.05 (CI = 95%).

## 3. Results

The sample consisted of 1014 Spanish people with asthma (587 females). The mean age of the participants was 43.2 (SD: 14.7, range: 15–69; Mo: 40). Characteristics of the participants are exhibited in Table 1.

Total PA of participants (MET·min/week) is presented in Table 2. All variables showed significant differences, except smoking. It was found that men, people with higher education level, those not married and not living as a couple, and those who drank alcohol, were more physically active. In relation to age, there were significant differences between participants aged 30–60 and over 60 with those under 30, the youngest group being the most active. However, when Pearson Correlation was employed, a low negative association was observed between age and weekly volume of PA (r= −0.106; *p* = 0.001). According to BMI, obese participants were significantly less physically active than people with normal weight and those overweight. Standard deviations reveal a high variability based on very low values in many participants.

In Table 3, PA was grouped into low, moderate and high level. The variables sex, age, education level, marital status, living together, alcohol intake and BMI showed significant differences. Post hoc analyses indicated significant differences between sex and alcohol consumption and high level of PA, with a higher percentage of men and those who drank alcohol. In relation to age, the significant differences were in high PA level between those under 30 years (29.3%) and those over 60 years (12.7%). There were also significant differences in low level of PA in relation to marital status and living as a couple, those who were married or were living as a couple having a higher percentage of low level. In relation to education, the significant differences were between those with the first period secondary or less achieved, and those with a tertiary level of education. According to BMI, no significant differences were found within subgroups. However, the percentage of obese participants with a low level of PA (44.9%) was significantly higher than the percentage of those with a high level of PA (12.8%). Considering the whole sample, a moderate level of PA was the most frequent (47.7%), and a high level of PA the least (20.7%).

The risk for low level of PA (<600 MET·min/week) was significantly higher in those with level A of education (OR = 1.470; 95%CI 1.055–2.048; *p* = 0.005) and those with higher BMI (OR = 1.040; 95%CI 1.012–1.069). Living together (OR = 1.141; 95%CI 0.642–2.029), having education level B (OR = 1.236; 95%CI 0.850–1.879), being older (OR = 1.005; 95%CI 0.994–1.016), being alcohol consumer (OR = 0.815; 95%CI 0.599–1.110) and being current smoker (OR = 1.150; 95%CI 0.815–1.624) revealed higher probability of having a low level of PA, but differences were not statistically significant (*p* > 0.05). The probability of having a low level of PA was reduced in men (OR = 0.774; 95%CI 0.580–1.031), past smokers (OR = 0.720; 95%CI 0.506–1.023) and/or unmarried participants (OR = 0.764; 95%CI 0.444–1.420). Nevertheless, these differences were not significant (*p* > 0.05).

## 4. Discussion

The results of this study show an average of 2228.9 MET·min/week of PA in adults with asthma residing in Spain. This amount of PA is higher than the minimum recommended by the Centres for Disease Control and Prevention (CDC) [35] and the World Health Organization [36], which established a weekly volume of at least 600 MET·min. Nevertheless, 31.6% of participants were found to have a low level of PA, which means a level under the recommendations. Moreover, the mean value of weekly PA in this investigation is lower than the volume observed in the international validation of the IPAQ-Short version carried out in 12 different countries with a total of 957 participants, which determined a mean value of 2514 MET·min/week in healthy adults [30].

When differences by sociodemographic characteristics were considered, the present study showed a lower total amount of PA in women, those older than 30, those without tertiary level of education, those married, those living with a couple, those who did no drink alcohol, and obese participants. This may be owing to a reduced quality of life in older people and in people with a lower education [37], potentially owing to poorer asthma control, which may be improved by increasing PA levels.

In 2015, Gerovasili et al. carried out a study to analyse levels of PA in 19,978 adults (from 18 to 64 years of age) in 28 different European nations. In Spain, an average of 2166 MET·min/week was found. However, the average in Southern Europe was lower than in the countries in Western Europe (2373 MET·min/week) and Northern Europe (2449.75 MET·min/week) [38]. The present study shows an average in Spanish asthmatics slightly higher than the Spanish average in the study of Gerovasili et al. (2228.9 MET·min/week vs. 2166 MET·min/week). This difference may be explained by the presence of disease not being considered in the study of Gerovasili et al.

It should be highlighted that most participants used in investigations of PA in asthmatics are children or adolescents. This could be due to the higher prevalence of asthma in children (14%) [16] in comparison to adults (4.3%) [3]. This is the first Spanish representative study that establishes the levels of PA in adults with asthma analyzing differences by gender, age, marital status, living together, education level, BMI, smoking habits and alcohol consumption.

Nevertheless, in 2001 Chen et al. analysed energy expenditure (EE) (kcal/kg·day) in leisure activities according to asthma and potential determinants. They found that EE was higher in men, in normal weight participants, in non-smokers and in men who did not drink alcohol (in the case of women, EE was higher in those who drank) [23]. This concurs with our study, where PA levels are higher in men and in normal weight asthmatics but differs in relation to tobacco consumption because there were no significant differences in PA levels between current smokers, past smokers and non-smokers. According to age, Chen et al. found that younger asthmatics were more active than non-asthmatics. However, among older asthmatics, EE were lower than in non-asthmatics [23]. The present study does not compare asthmatics with non-asthmatics, but shows that that total amount of PA is significantly higher in younger asthmatics (2699.0 MET·min/week) than in middle age asthmatics (2243.2 MET·min/week) and older adults with asthma (1619.3 MET·min/week) residing in Spain.

Regarding marital status and cohabiting, the present study found a higher percentage of low level of PA among those married and/or living together. In a German longitudinal study with a 19- year follow-up, results showed that cohabiting and being married were associated with reduced weekly PA in comparison with singles [39]. These results are in line with our findings. A possible explanation for the negative impact of relationships on PA level could be that people want to spend more time with their significant other or have wider family commitments and therefore have less time to be physically active. Similarly, a study carried out in adults from Pamplona (Spain) revealed higher risk of sedentarism among married men and women [40].

In relation to alcohol intake, a higher percentage of participants who do not reach PA recommendations was found among those who did not drink in comparison to those who drank (37.6% vs. 28.8%). However, the percentage of alcohol consumers with high level of PA was higher than non-consumers (15.5% vs. 23.1%). Actually, sport per se is associated with high alcohol consumption. Kingsland et al. [41] found that sport clubs alcohol management practices, such as service of alcohol, happy hour promotions and alcohol-only awards, were associated with higher alcohol consumption among club members.

Regarding BMI, Conroy et al. [21] evaluated 125 adults (≥21 years old) with asthma in New York and Denver, and they found that obese (≥30 kg/m2) or overweight (25–30 kg/m^2^) individuals engaged in lower moderate-vigorous PA than those with normal weight (35.7, 39.9, 46.2 min/day; *p* = 0.09). This partially concurs with our results, which shows significant differences between BMI groups. A total of 44.9% of obese asthmatics reported low levels of PA, with only 12.8% showing a high level. In this way, Russell et al. [42] reported that only in participants with normal weight (BMI < 25) did PA exhibit a positive correlation with asthma symptoms. Therefore, it is necessary to take BMI into account due to the fact that a high BMI is related to other chronic diseases like cardiovascular diseases, diabetes or cancer, and these could be involved in lower PA levels.

Recently, Sánchez-Castillo et al. carried out a similar study in Spanish people with Chronic Obstructive Pulmonary Disease (COPD) [43]. The total PA volume in COPD patients was lower (1684.8 MET·min/week) than the total amount of PA found in the asthmatics of the present study (2228.9 MET·min/week). This may be due to the progressive and minimally reversible obstruction that is found in people with COPD, while in asthmatics the obstruction should be variable and reversible [44]. However, when the classification level of PA was analysed, a significant percentage of participants in both the COPD study and the present study demonstrated a moderate level of PA (47.5% in COPD vs. 47.7% in asthma). On the contrary, people with a high level of PA were slightly more frequent in the present study (20.7%) than in the COPD study (14.6%) [43]. When differences by sociodemographic characteristics were considered, both studies found that PA was higher in men, younger adults, in people with normal weight, and in people who drank alcohol. However, the present study also showed higher levels of PA in those married and/or living together and in those with tertiary education. Regarding tobacco consumption, both studies found no significant differences in PA between current, past and non-smokers. A possible explanation of these results could be that PA could act as an ally and mitigate the damage caused by cigarette smoking in respiratory health. Otherwise, the prevalence of smoking (current and past) was higher in people with COPD than in asthmatics (67.0% vs. 49.31%).

Regarding the risk for low level of PA, it was found that higher BMI and lower level of education increased the risk of not achieving PA recommendations in asthmatics. Similarly, an American study about correlates of American adults’ PA patterns showed that older ages (≥60 years), female sex, higher BMI and history of chronic disease were significantly associated with lower odds of being more active [45]. Comparisons should be considered carefully, as the present analysis only included Spanish asthmatics. Another cross-sectional study in the Portuguese population revealed that moderate PA was a predictor of controlled asthma in men (OR = 1.84; 95%CI: 1.02–3.30) and vigorous PA doubled the risk of uncontrolled asthma in women (OR = 1.94; 95%CI: 1.13–3.35) [46].

Considering all data, it is important to carry out therapeutic interventions in order to increase PA levels in people with asthma. The main physical therapies for asthmatics are inspiratory muscle training [47], breathing exercises [48] and physical training [14]. Delgado et al. [47] found higher improvements in respiratory muscle strength and functional capacity in asthmatics who were involved in inspiratory muscle training. In relation to breathing exercises, Santino et al. [48] carried out an intervention review of breathing exercises: yoga, breathing retraining and Buteyko. The results revealed a higher change in quality of life (mean difference (MD) up to 3 months = 0.42; MD over 6 months = 1.34), better control of asthma symptoms (MD 0.15 up to 3 months), better control of hyperventilation symptoms (MD 3.22 up to 3 months) and better lung function up to three months (FEV_1_% predicted; MD: 6.88). Carson et al. [14] studied physical training for asthma and found significant improvement in maximum oxygen intake, positive effect on quality of life and identified no adverse effects, which means physical training was well tolerated by asthmatics. Therefore, physical therapies could be an important non-pharmacological treatment for people with asthma. Principal strengths of this paper include the use of a representative sample of asthmatics (15–69 years) residing in Spain and the use of a verified and internationally accepted questionnaire to evaluate levels of PA. Nevertheless, these results should be considered in the light of its restrictions. Although the IPAQ is a reference tool to establish a population’s level of PA, it is important to underline that it is self-reported, so people could over- or underestimate their PA level. In the same way, diagnosis of asthma was self-reported, too. Another potential limitation is the lack of classification of severity of asthma because data was not available in the survey. Data about possible therapeutic interventions followed by participants were not available, but should be considered in future studies. In addition, as this was a cross-sectional analysis and not a randomized controlled trial, causality cannot be proven, but only correlation. In future studies it will be advisable to measure PA levels objectively by employing accelerometers and to consider the severity of the asthma.

## 5. Conclusions

Approximately three out of ten Spanish people with asthma exhibited a low level of PA, not achieving PA recommendations. Therefore, PA programs should be executed to make people aware of its benefits in asthma control, and these intervention programs should target women, people of at least 30 years, married people and/or those living in a couple, those with a level of education equal or under the first period of secondary, those who do not consume alcohol, and people who do not have a normal weight. Moreover, long term PA programs are needed to increase PA levels and/or reach PA recommendations, so the activities involved in these programs should motivate the participants to sustain PA over time. In this way, asthmatics could make their life better.

## Figures and Tables

**Table 1:** Sample characteristics.

Total (*n* = 1014)		N	%
Sex	Females	587	57.89
	Males	427	42.11
Age	<30	205	20.22
	30–60	636	62.72
	≥60	173	17.06
Education level	Level A	466	45.96
	Level B	195	19.23
	Level C	353	34.81
Marital Status	Married	509	50.20
	Not Married	505	49.8
Living in couple	Yes	547	53.94
	No	460	45.36
	Missing	7	
BMI	Underweight	28	2.82
	Normal weight	420	42.34
	Overweight	323	32.56
	Obesity	221	22.28
	Missing	22	
Smoking	Current	235	23.18
	Past	265	26.13
	Never	514	50.69
Alcohol last 12 month	Yes	692	68.24
	No	322	31.76

N: sample size; %: percentage; Level A: ≤first period secondary; Level B: second period secondary and post-secondary (not tertiary); Level C: tertiary; BMI: body mass index.

**Table 2:** Physical Activity in Met·min/week by characteristics of the sample.

		*n*	Av	SD	Med	*p*	ES
Sex	Females	587	2019.5	3021.8	1188.0	0.005	0.007
	Males	427	2516.8	3624.1	1386.0		
Age	1. <30 ^2,3^	205	2699.0	3109.5	1470.0	<0.001	0.059
	2. 30–60 ^1^	636	2243.2	3521.7	1350.0		
	3. ≥60 ^1^	173	1619.3	2470.6	924.0		
Education level	1. Level A ^3^	466	2168.3	3808.6	1039.5	0.001	0.014
	2. Level B	195	2121.5	2552.0	1386.0		
	3. Level C ^1^	353	2368.3	2913.5	1386.0		
Marital Status	Married	509	2089.5	3582.4	1039.5	0.001	0.011
	Not Married	505	2369.4	2977.5	1386.0		
Living in couple	Yes	547	2177.0	3615.5	1173.0	0.010	0.007
	No	460	2301.7	2898.5	1386.0		
Smoking	Current	235	2512.5	4104.9	1188.0	0.890	0.000
	Past	265	2074.5	2969.2	1386.0		
	Never	514	2178.9	3030.2	1386.0		
Alcohol (last 12 months)	Yes	692	2378.3	3312.9	1386.0	0.001	0.011
	No	322	1907.9	3242.5	1011.0		
BMI	1. Underweight	28	2376.2	4050.9	1014.7	<0.001	0.035
	2. Normal weight ^4^	420	2439.4	3071.4	1386.0		
	3. Overweight ^4^	323	2454.0	3726.3	1386.0		
	4. Obesity ^2,3^	221	1599.8	2870.2	693.0		
Total		1014	2228.9	3296.4	1386.0		

*n*: Sample size; Av: Average; SD: Standard Deviation; Med: median; Level A: ≤first period secondary; Level B: second period secondary and post-secondary (not tertiary); Level C: tertiary; Superscripts indicate significant differences between groups; *p*-values were based on Mann- Whitney U test and Kruskal Wallis H test; ES: Effect size was based on eta squared for Mann-Whitney U test and epsilon squared for Kruskal Wallis H test. Statistical significance: *p* < 0.05.

**Table 3:** Classification of physical activity (PA) level following International Physical Activity Questionnaire (IPAQ) guidelines, by characteristics of the sample.

		*n*	PA Level				
			Low	Mod	High	*p*	*V*
Sex	a Females	587	198(33.7)	287(48.9)	102(17.4) ^b^	0.007	0.099
	b Males	427	122(28.6)	197(46.1)	108(25.3) ^a^		
Age	a <30	205	53(25.9)	92(44.9)	60(29.3) ^c^	0.001	0.094
	b 30–60	636	200(31.4)	308(48.4)	128(20.1)		
	^c^ ≥60	173	67(38.7)	84(48.6)	22(12.7) ^a^		
Education level	a Level A	466	171(36.7) ^c^	208 (44.6)	87(18.7)	0.017	0.077
	b Level B	195	59 (30.3)	95 (48.7)	41 (21.0)		
	c Level C	353	90 (25.5) ^a^	181 (51.3)	82 (23.2)		
Marital Status	a Married	509	182(35.8) ^b^	237(46.6)	90(17.7)	0.005	0.102
	b Not Married	505	138(27.3) ^a^	247(48.9)	120(23.8)		
Living in couple	a Yes	547	192(35.1) ^b^	254(46.4)	101(18.5)	0.019	0.089
	b No	460	127(27.6) ^a^	224(48.7)	109(23.7)		
Smoking	Current	235	79(33.6)	100(42.6)	56(23.8)	0.127	0.060
	Past	265	73(27.5)	143(54.0)	49(18.5)		
	Never	514	168(32.7)	241(46.9)	105(20.4)		
Alcohol (last 12 m)	a Yes	692	199(28.8) ^b^	333(48.1)	160(23.1) ^b^	0.003	0.107
	b No	322	121(37.6) ^a^	151(46.9)	50(15.5) ^a^		
BMI*	a Underweight	37	9(32.1)	14(50.0)	5(17.9)	<0.001	0.112
	b Normal weight	419	116(27.6)	201(47.9)	103(24.5)		
	c Overweight	320	89(27.6)	163(50.5)	71(22.0)		
	d Obesity	227	102(44.9) ^d^	96(42.3)	29(12.8) ^d^		
Total		1014	320(31.6)	484(47.7)	210(20.7)	<0.001	

Values are Frequency (%). *n*: Sample size; Mod: moderate; Level A: ≤first period secondary; Level B: second period secondary and post-secondary (not tertiary); Level C: tertiary; *V*: Cramer’s V. Superscripts indicate significant differences between groups; Statistical significance: *p* < 0.05.
